# Policy implications of the 2020–22 Australian study of mental health and wellbeing

**DOI:** 10.1177/00048674241292961

**Published:** 2024-11-07

**Authors:** Maree Teesson, Harvey Whiteford, Marlee Bower, Scarlett Smout, Philip Burgess, Meredith G. Harris, Jane Pirkis, Sandra Diminic, Andrew Baillie, Tim Slade, Cath Chapman

**Affiliations:** 1 The Matilda Centre for Research in Mental Health and Substance Use, The University of Sydney, Sydney, NSW, Australia; 2 The University of Queensland, Brisbane, QLD, Australia; 3 Queensland Centre for Mental Health Research, Brisbane, QLD, Australia; 4 Centre for Mental Health and Community Wellbeing, The University of Melbourne, Melbourne, VIC, Australia; 5 Sydney School of Health Sciences, Faculty of Medicine and Health, The University of Sydney, Sydney, NSW, Australia

**Keywords:** Government, mental health, planning, policy

## Abstract

The objective of this paper is to summarise the policy implications of key findings from the 2020–22 Australian National Study of Mental Health and Wellbeing (NSMHWB). We provide an analysis of policy implications of four papers in this issue of the journal from the 2020–22 NSMHWB (*N* = 15,893) and the 2007 NSMHWB (*N* = 8841). The 2020–2022 NSMHWB reported a lifetime prevalence rate of common mental disorders of 40.2% (95% confidence interval [CI] = 39.2–41.3) and 12-month prevalence rate of 20.2% (95% CI 19.5–21.0). Overall, adult Australians were significantly more likely to experience a 12-month mental disorder in 2020–22 compared with 2007, with the change most striking in among those aged 16–24 years (odds ratio [OR] 1.2, 95% CI 1.1–1.3). Individuals aged 16–24 years in 2020–22 were significantly more likely to experience a 12-month anxiety disorder (OR 2.9, 95% CI = 2.3–3.7, depressive disorder (OR 2.8 95% CI = 2.1–3.9) or comorbidity (relative risk [RR] = 1.4, 95% CI = 1.2–1.7) compared with those aged 16–24 years in 2007. In 2020–22, the proportion of Australians who had experienced suicidal ideation, suicide plans and suicide attempts in the past 12 months was 3.3%, 1.1% and 0.3%. Under half (46.5% 95% CI 44.1–48.8) of adults with a 12-month mental disorder sought treatment. Mental disorders remain an endemic feature of Australia’s overall health landscape and appear to be increasing, especially in younger cohorts. While service use rates have improved over time, there is still some way to go. Epidemiological surveys such as the 2020–22 NSMHWB are important for understanding changing prevalence and the population not accessing services. Innovative prevention and treatment strategies will be needed to address the increasing rates of disorders in younger Australian adults. Equally innovative and bold policy responses will be essential.

## Introduction

The development of an effective mental health response in Australia must be built on the best possible data, reflecting the needs of all Australians. In Australia, we have three national mental health survey initiatives – the National Study of Mental Health and Wellbeing (NSMHWB), the Australian Child and Adolescent Surveys of Mental Health and Wellbeing and the Surveys of High Impact Psychosis. The NSMHWB – which provide population-level data on service use and the prevalence of mood, anxiety and substance use disorders in Australians aged 16–85 years – are the focus of this paper (Andrews et al., 1999, 2001; Slade et al., 2009, in preparation). The NSMHWB use validated measures, rigorous sampling methods and high-quality data collection. This paper outlines some of the policy implications of the most recent NSMHWB (2020–22).

One of the most important features of the Australian NSMHWB is that they are representative of the Australian adult population. Without such general population data, we are reliant on information limited to populations who present for health care, or who can raise their needs through advocacy. Even though the last decade has seen significant improvements in the quality of data available through health systems and early monitoring, there remain limitations. For example, the world-first National Suicide and Self-harm Monitoring System (NSSHMS) (Australian Institute of Health and Welfare [AIHW], 2023a) collates data on self-harm but only captures episodes of self-harm which involve treatment by hospital or ambulance staff. Those who do not engage with the health system are not captured, rendering certain groups and demographics invisible. Indeed, much of the data available to policymakers comes only from people who contact services.

The population focus of the NSMHWB is particularly important for the national interest, given the tendency for media and advocacy in Australia to focus on access to care and long wait times for those who are already seeking help. While these are both vital concerns for a well-functioning health system, what is much less obvious but also a critical issue, is the significant number of individuals who have not yet, or may never, seek treatment. The impact on individuals is profound. Lack of treatment can result in a lifetime of lost productivity, poor outcomes and impacts on life functioning. These impacts also extend to people who care for and support the individual and more generally to lost societal productivity (Productivity Commission, 2020). The delay or lack of service access is the result of many factors and can differentially impact across age cohorts. Population studies allow us to understand the magnitude of mental disorders in the whole population and especially among those who have not sought help.

## Key findings of the two prior NSMHWB and resulting policy reforms

There have been three NSMHWB since 1997. Repeating surveys allows an understanding of changes in prevalence and treatment access rates over time. The Australian Bureau of Statistics undertook Australia’s first two NSMHWB in 1997 (Andrews et al., 1999) and 2007 (Slade et al., 2009). These surveys and the reports they produced have had a major impact on our understanding of mental health in Australia. First, the 2007 survey replicated the 1997 NSMHWB findings that one in five Australians meet criteria for a prior 12-month mental disorder and that almost half of Australian adults will experience a mental illness in their lifetime. These prevalence data have enabled the estimation of the significant burden of disease attributable to mental disorders (AIHW, 2023c). Second, the 2007 and 1997 NSMHWBs found that most people did not seek or receive treatment for their mental disorders from a health professional (Andrews et al., 2001; Slade et al., 2009). Finally, these surveys demonstrated that young adults experienced higher rates of mental disorders compared with other age groups, consistent with international data (McGrath et al., 2023; Solmi et al., 2022).

Importantly, findings from these studies have been instrumental in shaping Australia’s policy responses to mental disorders. In response to the 1997 NSMHWB, the National Mental Health Strategy was expanded to include a broader focus. Prior to the national surveys, the focus had been on structural reform of public-sector specialised mental health services. These services focus on acute care and risk, especially for people with psychosis, and severe mood anxiety and personality disorders. The NSMHWB led to policy initiatives to expand service options for individuals with more common presentations of mental disorders and initial steps to improve access to care for youth. These initiatives include the Better Access to Psychiatrists, Psychologists and General Practitioners (GPs) (‘Better Access’) (Pirkis et al., 2022a). The consistent high prevalence of mental disorders in the first two NSMHWB has been a driving issue in the 55 high-profile public reports relevant to mental health published since the first survey 30 years ago, involving more than 55,000 submissions and 9000 witnesses (Francis et al., 2022). To summarise several of these reports, first the 2015 review of National Mental Health Programmes and Services (Department of Health and Aged Care, 2015) highlighted the barriers to high-quality care for people with mental disorders and the fragmentation of the mental health system, presenting a case for long-term reform. Second, the 2017 Fifth National Mental Health and Suicide Prevention Plan (Australian Department of Health, 2017) established a national approach for collaborative government action across Australia to improve the provision of better integrated mental health services. It continued to expand the scope of care for mental disorders across the population, including anxiety and depression. In 2020, the Productivity Commission Inquiry Report (Productivity Commission, 2020) focussed on the effect of mental health on people’s ability to participate and thrive in the community and workplace. The report focused on the broader impacts of mental disorders on the Australian economy and productivity, emphasising that reform of the mental health system would produce large social and economic benefits, particularly for people’s quality of life. These benefits were valued at up to $18 billion with an additional annual benefit of up to $1.3 billion from increased economic participation. In 2021, the National Mental Health and Suicide Prevention Plan (Australian Government, 2021) articulated a plan for how the Australian Government will support mental health and suicide prevention for all Australians, closely followed by the National Mental Health and Suicide Prevention Agreement in 2022 (Federal Financial Relations, 2022) which set out the shared intention of the Commonwealth, state and territory governments to work in partnership to improve the mental health of all Australians.

Since 2007, there have been changes to Australia’s mental health service system: primary mental health care programmes have grown (e.g. headspace, Better Access); telephone and online services have expanded (e.g. Head to Health); new service models have emerged for people with enduring problems (e.g. the National Disability Insurance Scheme) and new services have been launched for people with problems that are too severe for primary care services (e.g. Head to Help). While none of these initiatives have been without some criticism, they are all responses to the recognised need to enhance the mental health system. There have also been calls for increased investment in the prevention of and early intervention with mental disorders, as well as efforts to address the social determinants of mental health.

## The 2020–2022 NSMHWB

More than 13 years after the 2007 NSMHWB, the Australian Bureau of Statistics carried out a third national mental health survey. The 2020–22 NSMHWB is the largest survey to date and is a component of the wider intergenerational Health and Mental Health Study funded by the Australian Government Department of Health. The 2020–22 NSMHWB comprised 15,893 participants from two cohorts. The first cohort provided data from December 2020 to July 2021, and the second cohort provided data from December 2021 to October 2022. The sample represents all usual residents in Australia aged 16–85 years living in private dwellings in urban and rural areas across all states and territories. Very remote parts of Australia and discrete Aboriginal and Torres Strait Islander communities were not included. The 15,893 fully responding households represented a response rate of 52%. Details of the sampling framework, complex survey design and administration of the survey are provided by Slade et al. (2024b).

The 2007 NSMHWB (Slade et al., 2009) was used for comparison to examine changes in the intervening 13-plus years. Detailed comparisons between the instrumentation and methods of the 2007 and 2020–22 surveys are provided in Slade et al. (2024b). Papers in this issue of the *Australian and New Zealand Journal of Psychiatry* provide methodology and key findings on prevalence (Slade et al., 2024b), comorbidity (Sunderland et al., in preparation), suicide (Arya et al., 2024) and service use (Harris et al., in preparation). The significant policy implications are covered in this paper.

## Mental disorders remain highly prevalent

The 2020–22 NSMHWB found that 1 in 5 people (20.2%) experienced a mental disorder in the last 12 months (Slade et al., 2024b). In the 2020–22 NSMHWB, anxiety disorders were the most common mental disorder class. Among the anxiety disorders, social anxiety disorder, post-traumatic stress disorder and obsessive-compulsive disorder were the most prevalent. Anxiety disorders were more than twice as common as depressive disorders, and almost five times as common as substance use disorders.

To date, there has been less policy focus on anxiety disorders. Stigma and poor mental health literacy have been identified as major barriers to the identification and treatment of anxiety and depressive disorders. Over the last 13 years, there have been concerted efforts to address these barriers, but this work has focussed on depression and to a lesser extent anxiety disorders (National Mental Health Commission, 2024). Given the high prevalence of anxiety disorders in 2020–22 NSMHWB, Australian efforts focussed on this class of disorders are an imperative.

Overall, the 2020–22 survey found that mental disorders were most common in young people aged 16–24 years (Slade et al., 2024b). This age group also saw the most dramatic increase in prevalence over time. In 2007 NSMHWB, the overall rate of prior 12-month mental disorders in young people was 26.4%; by 2020–22, this has increased to 38.8%. Australians aged 16–24 years were significantly more likely to experience a 12-month anxiety disorder (odds ratio [OR] 2.7), depressive disorder (OR 2.8) or experience more than one diagnosable mental disorder (relative risk [RR] = 1.44) in 2020–22 compared with those aged 16–24 years in 2007.

The increased rates of mental disorders in younger Australians do raise questions about whether these are true changes in prevalence or an artefact. Recent studies using longitudinal cohorts and age period cohort analysis add weight to the veracity of findings of cross-sectional studies like the 2020–22 NSMHWB. Other studies for example, have found that the prevalence of common mental disorders and psychological distress has increased across cohorts of adolescents (Botha et al., 2023; Halladay et al., 2024a; Slade et al., 2024a). Importantly, observed changes in distress in these studies did not appear to be explained by changes in how adolescents potentially interpreted and responded to the distress questions over time. What is difficult to know between cohorts is the counter-factual; when comparing older and younger cohorts at the same age would we expect the older cohort to indicate the same prevalence of mental disorders as the younger cohort if they had experienced the same broader contextual and environmental circumstances? Older cohorts were not exposed to social media, constant negative news streams, climate crises and a global pandemic. Older cohorts were exposed to different challenges, but it is possible that these exposures were not as strongly associated or related to mental health outcomes. Age cohorts will always experience different contexts and we would argue that we should indeed take the increase in prevalence in younger Australian adults seriously. Why are younger Australian adults experiencing more mental disorders than previous cohorts? Some hypotheses include (1) shifts in how people spend their time and connect with each other so today’s youth are more disconnected and disaffiliated; (2) shifts in lifestyle factors more broadly (i.e. substance use, sleep, exercise, screen time and nutrition); (3) increasingly stressful/anxiety-inducing environments related to climate, economic and political and health crises and (4) financial insecurity, work-related pressures, expectations and cultures (‘hustle culture’) (Halladay et al., 2024b; Krokstad et al., 2022; Patalay and Gage, 2019; Smout et al., 2023). While there are several hypotheses, none have consistently been identified as a main driver of the increased prevalence in young Australians.

Many of the participants in the 2020–22 NSMHWB completed their survey during the COVID-19 pandemic. The pandemic itself has demonstrated that global threats have significant impacts on the mental health of the population (Santomauro et al., 2021). This impact was experienced disproportionately by women, youth and some disadvantaged and cultural groups (Bower et al., 2023).

Since the first two NSMHWB, substantial evidence has grown for the impact of social determinants of health and mental health, but there has been disappointingly little emphasis on which factors are important and how they can be modified by policy interventions (Batterham et al., in press). A recent Australian policy analysis synthesised research and suggested that increasing youth income support payments, supporting access to tertiary education and addressing social disconnection are actionable policy responses to address the social determinants of mental health among young Australians (Australia’s Mental Health Think Tank et al., 2023).

The 2020–22 NSMHWB findings further underscore the need for the consideration of suicidal ideation, suicide plans and suicide attempts and not just suicide in policy development (Australian Government, 2021). The 2020–22 NSMHWB demonstrated increases in rates of suicidal ideation and suicide plans for males and increases in rates of suicidal ideation for those aged 16–24 years (Arya et al., 2024). Two-fifths of those who attempted suicide during the previous 12 months did not use health services following their attempt. The new strategy should also consider self-harm more broadly, given the significant overlap between suicide attempts and self-harm without suicidal intent. Suicidal ideation, suicide plans, suicide attempts and self-harm without suicidal intent provide opportunities for intervention that may ultimately bring down the suicide rate.

## Mental disorders often co-occur with other mental disorders

The NSMHWB 2020–22 confirmed that co-occurrence between mental and substance use disorders remains a significant challenge for the Australian population, with 46% of people with a past 12-month mental or substance use disorder in 2020–22 experiencing two or more diagnosable conditions. Those with higher levels of psychological distress, higher service use and higher rates of suicidality were at greater odds of experiencing co-occurring disorders, with dose–response relationships appearing between number of co-occurring disorders and the experience of distress, service use and suicidality (Sunderland et al., in preparation). Overall, as noted by Sunderland et al. (in preparation), the experience of co-occurring disorders is endemic.

Since the 2007 NSMWHB, there have been significant advances in treatment of co-occurring mental and substance use disorders (Marel et al., 2022). The Australian comorbidity guidelines and associated training materials are a government-supported initiative to increase the skills required to provide treatment and care for people experiencing more than one mental or substance use disorder, including those with extensive trauma histories (Marel et al., 2022). These outstanding practice initiatives to address comorbidity have not been matched by a clear policy focus. The last National Comorbidity Initiative was over 20 years ago (AIHW, 2005). The prevalence of co-occurring disorders in Australia and elsewhere (Halladay et al., 2022) has not declined since the previous survey conducted in 2007. In fact, rates for those aged 16–24 years have increased by 44% (Sunderland et al., in preparation). Global and local research shows that mental health and substance use disorders commonly co-occur with physical health conditions, so future research should examine this within the NSMHWB (Halstead et al., 2024; Momen et al., 2020).

## Implications for prevention

The rising prevalence of mental disorders in young people demonstrated by the NSMHWB highlights the need for a broad prevention approach. Given that prevention of common mental disorders can be effectively delivered across multiple settings including school, workplaces and online (Cuijpers et al., 2021), policy reform needs to be directed towards ensuring that people have access to evidence-based prevention. The best evidence to date is that prevention ‘works’ in reducing the incidence of depression, by about 19% (Batterham et al., in press; Cuijpers et al., 2021). While this would potentially create a significant impact by reducing the prevalence of common mental disorders, new models for prevention also need to be tested embedding ongoing low-intensity elements such as health promotion and monitoring across the lifespan (Grummitt et al., 2023). There may also be lessons for the prevention of common mental disorders informed by successes in the prevention of chronic diseases such as cardiovascular disease and diabetes, where policy has focussed on addressing continuous lifestyle change (The Heart Foundation, 2024).

## Treatment rates have improved but remain low

A major advantage of repeated cross-sectional studies is the ability to study changes in treatment rates over time. The service use paper in this issue by Harris et al. (in preparation) reports several important changes in Australians’ use of services for mental health from 2007 to 2020–22. In 2020–22, more than 17% of adults used services for mental health, an impressive increase of 46% compared 2007. This shift was driven by increased rates of consultation with psychologists and, to a lesser extent, with other (non-medical) mental health professionals, and GPs. However, these changes were not experienced uniformly across all clinical and population sub-groups.

Overall in 2020–22, 46.5% of people with 12-month mental or substance use disorders had used services in the past year. This is an important increase from 37.5% in the 2007 NSMHWB. Equally important is that the expected service gradient according to severity of disorder was observed (mild 23%, moderate 48% and severe 69%) (Harris et al., in preparation). However, the 2020–22 rates still fall short of targets proposed through the National Mental Health Service Planning Framework (67% overall, and 50%, 80% and 100% for mild, moderate and severe disorders, respectively) (AIHW, 2024).

The landscape of mental health treatment provision has also changed over the past 13 years, especially with the introduction of ‘Better Access’. Policy changes have clearly led to increased rates of consultation with psychologists (123%), other (non-medical) mental health professionals (64%) and GPs (53%). Importantly, since 2007, younger adults made greater use of services and of GPs in particular, but overall rates of service use remain persistently low in this age group. Service use by men with disorders also remains low (38.5% vs 52.3% for women) and the adequacy of our system to address the care needs of males remains a concern. Changes in service use varied across population sub-groups. People who do not meet the criteria for mental disorders but may have subthreshold symptoms (Bobevski et al., 2017; Harris et al., in preparation) had increased rates of consultation with psychologists and other mental health professionals.

Service use for young people aged 16–24 years with disorders improved between 2007 and 2020–22, especially among males. Overall, young adults with disorders went from having lower rates of service use in 2007 compared with their middle-aged counterparts, to having comparable rates in 2020–22. These patterns are consistent with observations from other data sources showing increases over time in the use of primary mental health care programmes by young people (Harris et al., 2010, headspace, 2019, 2021; Pirkis et al., 2022b). However, fewer than half (46.4%) of those aged 16–24 years who met the criteria for a mental disorder in the past 12 months accessed treatment (Harris et al., in preparation). This, coupled with the high prevalence of mental disorders among this age group, suggests that an even greater focus on youth mental health is warranted.

There has been a major focus on youth mental health service provision over the last two decades, including the development of key national programmes such as ***headspace***. However, high and increasing rates of mental disorders in those aged 16–24 years, coupled with fewer than half (46.4%) accessing any treatment means this remains a significant policy issue. Furthermore, given that peak age of onset of anxiety, depressive and substance use disorders is in adolescence and young adulthood, early intervention must target these ages and prevention efforts must target those at risk before disorders are established (Batterham et al., in press; Grummitt et al., 2023). Prevention and treatment services for younger Australians require increased investment. It is increasingly recognised that youth mental health policy and service design benefit from the inclusion of perspectives and input from young people themselves, who are acutely aware of the issues impacting their mental health (Australian Government, 2024; Orlowski et al., 2015). However, care must be taken to ensure that youth participation is implemented in a meaningful way that involves young people from the outset and sustains a continuous partnership that is valued and prioritised.

## Expenditure on services

Spending on mental health-related services increased from $9.3 billion in 2016–17 to $11.6 billion in 2020–21. Overall, national spending increased from $418 per person in 2016–17 to $451 per person during 2020–21, which represents an average annual increase of 2%. However, overall, the mental health identified proportion of total health expenditure decreased in 2020–21 (to 7%, from 8% in 2019–20).

## The role of research and innovation

Research and innovation are critical to advancing the mental health of the whole community. Mental health research informs our understanding of how we can prevent mental disorders, what treatments and interventions are effective, how we can monitor and evaluate those treatments and interventions, how we organise and deliver mental health care and more. The performance of Australian research within the global mental health field is impressive (Scimago Journal & Country Rank, 2023). However, it is not funded at the scale needed for widespread impact.

When considering that 15% of the burden of disease is caused by mental health and substance use disorders (AIHW, 2023b), and when compared with other leading causes of burden of disease in Australia, the funding for mental health research and innovation in Australia is not adequate to meet current and future challenges. Most funding comes from Australia’s two largest health and medical research funders, the Medical Research Future Fund (MRFF) and the National Health and Medical Research Council (NHRMC). While mental disorders account for 15% of the burden of disease in Australia, only 7.4% of MRFF funding and 11.5% of NHMRC (2023) funding went towards mental health research (Department of Health and Aged Care, 2024).

The scale of mental health disorders in Australia requires innovative solutions. Research drives innovative solutions, but the lack of significant investment undermines the capacity to deliver the paradigm-shifting innovations that are required. In 2022, at the request of the Australian Government, the National Mental Health Commission (2022) developed a ***National Mental Health Research Strategy*** to strengthen the mental health research system. The strategy provides the principles to guide and support decision-makers such as funders and researchers. It outlines the actions for a system reform that will ensure mental health research enables and reflects the significant reforms occurring in the mental health system. The strategy calls for a national mental health alliance to lead the strategic direction of Australia’s mental health landscape, drive the creation of new evidence, facilitate innovation and bring together key stakeholders to seed evidence-based action throughout the system.

## Conclusion

The 2020–22 NSMHWB is a unique source of information from thousands of Australians. It helps us understand the impact of our investment in mental health on the Australian population and identify gaps in our approach. While overall health expenditure in Australia has grown, this has not translated into a proportionate increase in investment in mental health, despite evidence of consistent pressure on the health system and – in light of the data discussed herein – significant worsening of the mental health of Australians. With stagnant investment, we fail to deliver mental health care to over half of the population with common mental disorders.

The Productivity Commission’s inquiry into mental health found that pathways of care in Australia are not sufficiently person-centred (Productivity Commission, 2020). The 2020–22 NSMHWB underscores this and further demonstrates that improving access to care should remain a policy imperative. Primary care remains the main point of access to health care for people with mental disorders and consultations in this sector have increased (Harris et al., in preparation). However, Australia’s primary care system is under strain (Breadon et al., 2022) and access to care remains a concern in the 2020–22 NSMHWB. The issues are widespread and include workforce shortages and burnout (exacerbated by the COVID-19 pandemic), insufficient services, limited training and resources to improve education and awareness, out-of-pocket costs, social stigma and cultural and financial barriers (Australian Academy of Health & Medical, 2023). The whole community must be able to access and afford services and support in the right way, at the right time for them and at prices they can afford.

While improving access is critical, it will not improve Australia’s overall mental health unless the care provided aims to be adequate and effective at every stage for everyone. The 2020–22 NSMHWB demonstrates that mental health care coverage is uneven and not universally distributed. At a minimum, mental health care should be accessible, affordable and adequate to ensure the best outcomes. While there are positive signs since the last survey, increases in service use among young Australian adults being one, the gaps in adequate coverage, care and research investment remain significant.

The 2020–22 NSMHWB is a major investment that provides unique information to help guide effective responses to mental health in Australia. The results of the five main papers contained in this issue provide vital information on the scope and impact of common mental disorders in the general population, which ultimately can help guide planning and policy for a healthier future.

Finally, the wealth of data contained in the 2020–22 NSMHWB, coupled with the ability to compare findings to the 2007 survey, highlights the importance of continuing this important initiative in the future, ideally at narrower intervals.
